# Minimum Dietary Fat Threshold for Effective Ketogenesis and Obesity Control in Mice

**DOI:** 10.3390/nu17203203

**Published:** 2025-10-12

**Authors:** Jiawen Shou, Xingchen Dong, Fei Sun, Jia Li, Huiren Wang, Qing Ai, Michael Pellizzon, Ting Fu

**Affiliations:** 1 Pharmaceutical Sciences Division, School of Pharmacy, University of Wisconsin-Madison, Madison, WI 53705, USA; 2 Research Diets, Inc., New Brunswick, NJ 08901, USA; 3 University of Wisconsin Carbone Cancer Center (UWCCC), Madison, WI 53792, USA

**Keywords:** ketogenic diet, hepatic and intestinal ketogenesis, obesity

## Abstract

**Background/Objectives**: Ketogenic diets (KDs), defined by very low carbohydrate and high fat content, are widely studied for obesity and metabolic disease. However, KD formulations vary from 60–95% fat, leading to inconsistent induction of ketogenesis and variable outcomes. The fat threshold required for sustained ketosis, and the tissue-specific programs that mediate KD efficacy, remain unclear. **Methods**: We evaluated multiple KD formulations (80–95% fat) in C57BL/6J wild-type (WT) and diet-induced obese (DIO) mice. Plasma, hepatic, and intestinal β-hydroxybutyrate (BHB) were measured together with expression of ketogenesis and fatty acid oxidation genes. Body weight, adipose distribution, and liver morphology were assessed under both direct feeding and therapeutic settings. **Results**: In WT mice, only diets exceeding 85% fat induced robust ketogenesis, reflected by elevated BHB and hepatic upregulation of Cd36, Cpt1a, Acat1, and Hmgcs2. Moderate KDs (80–85%) failed to trigger ketosis and resembled high-fat feeding. In obese mice, an 80% KD lowered fasting glucose without reducing body weight, whereas a 90% KD promoted systemic ketosis, weight loss, and adipose reduction. Interestingly, hepatic transcriptional programs for fatty acid oxidation and ketogenesis were suppressed under 90% KD despite elevated BHB, suggesting reliance on substrate availability and peripheral utilization. In contrast, intestinal Hmgcs2 was strongly induced in both WT and DIO mice, with Oxct1 upregulated only in obesity, indicating local ketone production and consumption. **Conclusions**: These findings identify > 85% dietary fat as a threshold for sustained ketosis and highlight distinct liver–intestine contributions, underscoring ketogenesis as the central driver of KD’s anti-obesity benefits.

## 1. Introduction

Obesity affects nearly one in five adults worldwide and has emerged as one of the most pressing health challenges of our time. It is a major driver of type 2 diabetes, fatty liver disease, cardiovascular disorders, and several cancers [1]. Obesity is a complex metabolic disorder characterized by excess adiposity, insulin resistance, and systemic energy imbalance [1,2]. Its rise is closely linked to Western-style diets, which are rich in refined carbohydrates and saturated fats and promote energy intake that chronically exceeds expenditure [3,4]. A central feature of obesity, particularly in diet-induced obesity (DIO) models, is impaired coordination between glucose and lipid metabolism [5]. In the healthy state, tissues flexibly switch between carbohydrate oxidation during feeding and fatty acid β-oxidation during fasting [6]. This “metabolic flexibility” allows the organism to maintain glucose homeostasis while efficiently mobilizing fat stores when carbohydrate supply is limited [7]. In obesity, however, chronic nutrient overload and hyperinsulinemia bias tissues toward carbohydrate use, suppressing β-oxidation and limiting the capacity to generate and utilize ketone bodies [8]. As a result, excess fatty acids accumulate in the liver and adipose depots, exacerbating insulin resistance and perpetuating weight gain [9]. Restoring fat oxidation and re-engaging ketone metabolism has therefore become an important therapeutic goal. By increasing reliance on β-oxidation and ketogenesis, metabolic interventions may reduce glucose excursions, relieve hepatic lipid burden, and improve systemic energy balance [10,11]. This rationale underlies growing interest in ketogenic diets, fasting, and exercise as strategies to reverse DIO and reestablish nutrient flexibility.

Ketogenesis is a highly conserved pathway that occurs primarily in the liver, where fatty acids—derived directly from dietary triglycerides, or from adipose tissue lipolysis—are oxidized to acetyl-CoA and subsequently converted into ketone bodies such as acetoacetate, β-hydroxybutyrate (BHB), and acetone [12,13]. These metabolites enter the circulation and are utilized by extrahepatic tissues through ketolysis, with the brain as the predominant consumer because it cannot oxidize fatty acids directly, followed by the heart and skeletal muscle [12,14,15]. In these organs, ketones provide an efficient fuel source during fasting, prolonged exercise, or ketogenic diet feeding. Ketogenesis is tightly linked to fatty acid β-oxidation, as the latter supplies the acetyl-CoA substrate pool necessary for ketone synthesis [16]. While the liver is the classical ketogenic organ, producing large amounts of ketone bodies but with only limited capacity for ketolysis [17], emerging studies suggest that the intestine may engage in both ketogenesis and ketolysis [18], raising important questions about its role in local energy homeostasis and epithelial function. The concept of ketosis—a physiological state in which circulating BHB is elevated—reflects the systemic manifestation of ketone body production and has become a practical marker of whether ketogenic interventions, such as KD, fasting, or exercise, have effectively engaged this metabolic program [19,20].

Although ketones were once considered mere alternative fuels, it is now clear that they also function as signaling molecules with broad regulatory effects. In the brain, BHB provides a critical energy source under fasting conditions and has been linked to neuroprotection in epilepsy [21], Alzheimer’s disease [22], and traumatic brain injury [23]. In the heart, ketone utilization rises during heart failure, suggesting an adaptive mechanism to sustain cardiac energetics under stress [24]. Beyond these classic energy roles, BHB is also a potent inhibitor of class I histone deacetylases (HDACs), thereby modulating chromatin accessibility and transcriptional programs. For example, in the intestine, ketone bodies regulate stem cell fate, where BHB enhances Notch signaling via HDAC inhibition to promote intestinal stem cells (ISC) self-renewal, lineage decisions, and regenerative capacity, with ketogenic diet feeding augmenting repair after injury [18]. In cancer, ketogenic diets and BHB have been shown to suppress colorectal tumor growth [25]. Ketogenic diets also remodel the gut microbiota in both humans and mice, marked by depletion of *bifidobacteria* and suppression of intestinal pro-inflammatory Th17 cells, thereby linking diet-induced ketogenesis to mucosal immune regulation [26]. In addition, KD consumption increases serum levels of bile acid derivatives such as TDCA and TUDCA, contributing to reduced body weight and improved glucose control in mice [27]. Collectively, these findings illustrate that ketones act not only as fuels but also as versatile signaling molecules with effects spanning energy metabolism, tissue regeneration, cancer suppression, immune regulation, and host-microbiome interactions.

Given this broad functional spectrum, ketogenic interventions have attracted attention as potential therapies across diverse diseases. In the clinical setting, ketogenic diets are already established for refractory epilepsy and are being tested in neurological disorders, cancer, type 2 diabetes, and obesity [12,28]. Intermittent fasting, alternate-day fasting, and time-restricted feeding represent alternative strategies that achieve ketosis by reducing carbohydrate intake and increasing reliance on fatty acid oxidation [29,30]. Exercise similarly promotes fat mobilization and ketone body production, linking lifestyle factors to metabolic remodeling [31]. Exogenous ketone supplementation, such as BHB esters or salts, provides yet another approach to mimic ketosis without strict dietary adherence [32]. Despite these diverse strategies, the unifying principle is that increasing fatty acid β-oxidation and ketogenesis can provide metabolic benefits, whether by improving energy efficiency, reducing glucose excursions, or modulating cell signaling pathways.

However, important knowledge gaps remain. In particular, it is not fully understood how different KD compositions, ranging from moderate to extreme fat content, affect systemic metabolism and organ-specific programs. Most studies have focused on the liver as the canonical ketogenic organ, yet evidence now suggests that the intestine may also engage in ketone metabolism, potentially for functions beyond energy supply, such as epithelial renewal, barrier maintenance, and immune regulation [18,26,33]. Moreover, host metabolic state, lean versus obese, may dramatically alter the response to KD. While lean animals might engage hepatic transcriptional programs robustly, obese animals undergoing weight loss may shift the burden of lipid and ketone utilization to peripheral tissues [12]. These possibilities underscore the need to disentangle how diet composition and metabolic context shape liver and intestinal contributions to ketone metabolism.

In this study, we address these questions by examining two complementary experimental frameworks: direct feeding of KDs with varying fat content (80–95%) to wild-type (WT) mice, and therapeutic KD interventions (80–90% fat) in mice with diet-induced obesity. This design allows us to define the fat threshold required for systemic ketosis, characterize tissue-specific ketogenic responses, and compare how lean and obese metabolic states influence hepatic and intestinal ketone metabolism. Through this approach, we aim to clarify the division of labor between liver and intestine in ketogenesis and ketolysis and to provide insight into how KD composition and host context interact to shape systemic and local outcomes.

## 2. Materials and Methods

### 2.1. The Animals

WT male C57BL/6J mice (8 weeks old; Cat. #000664, Jackson Laboratory, Bar Harbor, ME, USA) were bred in-house and maintained under specific pathogen-free conditions at the University of Wisconsin-Madison. Mice were housed 3 per cage with ad libitum access to food and water, under a 12 h light/dark cycle in AAALAC-accredited facilities. Animals were randomly allocated into each groups and maintained for six weeks on one of the following diets: low fat diet (LFD, Research Diets #D12450K), high-fat diet (HFD, Research Diets #D12492), 80% KD (Research Diets #D03022101), 80% fat plus 5% carbohydrates (Research Diets #D06040601), 85% KD (Research Diets #D06040603YM), 90% KD (Research Diets #D16062902), or 95% KD (Research Diets #D22011404OM). Detailed diet formulations are provided in Table 1 and Supplementary Table S1. Mice on the indicated diets were sacrificed on day 39, and tissue harvest was performed between 1:00 and 3:00 p.m. at the end of the feeding period.

For the DIO studies, mice were first maintained on an HFD for 8 weeks and then switched to 80% KD, 80% fat + 5% carbohydrates, or 90% KD for an additional 6 weeks. DIO mice were sacrificed after 39 days of treatment with either 80% or 90% KD, and tissues were harvested between 1:00 and 3:00 p.m.

In direct feeding studies, all KD groups were compared to LFD-fed controls, whereas in therapeutic DIO studies, all KD groups were compared to HFD-fed controls.

For each experiment, n = 5 mice per group, with the biological unit defined as the individual mouse. Experiment design flows were shown in panel A of related figures and summarized in Table S2.

All experiments were independently repeated twice and approved by the Institutional Animal Care and Use Committee (IACUC) of the University of Wisconsin-Madison, in accordance with Association for Assessment and Accreditation of Laboratory Animal Care (AAALAC) International guidelines.

### 2.2. Hematoxylin & Eosin (H&E) Staining and Alcian Blue Staining

H&E and Alcian Blue staining were performed as described in our previous work [34,35]. Paraffin-embedded tissue sections were first deparaffinized and rehydrated by sequential treatment with Histo-Clear (three times, 5 min each), followed by graded ethanol washes (100%, 75%, 50%, and 25%; 5 min each) and finally rinsed twice in tap water (5 min each). For H&E staining, slides were exposed to hematoxylin solution (Ricca Chemical Company, Batesville, IN, USA, cat# 353732) for 45 s, rinsed in water, and then counterstained with eosin solution (Ricca Chemical Company, cat# 284532) for 45 s. For Alcian Blue/PAS staining, sections were incubated in 1% Alcian Blue (pH 2.5, prepared in 3% acetic acid; EMS, cat# 26026-13) at room temperature for 30 min, followed by hematoxylin counterstaining for 5 s and a brief 10 s incubation in 0.01 N HCl. After each staining procedure, slides were washed three times with water. Stained sections were dehydrated through 90% ethanol (two times, 2 min each) and 100% ethanol (two times, 2 min each), cleared in Histo-Clear (three times, 2 min each), and finally mounted in glycerol with coverslips sealed using nail polish.

### 2.3. Glucose and Ketone Body Measurement

To assess the effects of dietary interventions on circulating metabolites, blood glucose and ketone levels were monitored using a Contour glucometer (Ascensia Diabetes Care US Inc., Parsippany, NJ, USA) and a ketometer (i-SENS, Inc., Seoul, Republic of Korea), respectively. In addition, β-hydroxybutyrate (BHB) concentrations were quantified in liver, and intestinal tissue samples using a commercially available ELISA kit (Cayman Chemical, cat #700190). For liver and intestinal tissues, BHB was extracted using the extraction buffer provided in the kit. Samples were sonicated with 5-s pulses and 5-s intervals, repeated three times, followed by centrifugation at full speed at 4 °C for 20 min. The resulting supernatants were collected and divided into two portions: one for BHB detection and the other for protein quantification using the Pierce™ BCA Protein Assay Kit (Thermo Fisher Scientific, Waltham, MA, USA, cat. #23227). The concentration of BHB in tissues was normalized into protein concentration.

### 2.4. Metabolite Measurement

Metabolites such as total bile acids (TBA), triglyceride (TG) and non-esterified fatty acids (NEFAs) were measured in mouse serum, liver and intestinal tissue samples by Total bile acid assay kit (Diazyme laboratories, Poway, CA, USA, cat #DZ042A-K), Triglyceride kit (Thermo Fisher Scientific, Waltham, MA, USA, cat #TR 22421/2780-250) and non-esterified fatty acids kit (Bioassay systems, Hayward, CA, USA, cat # 434-91795 and 436-91995) according to manufacturer’s instructions. Serum samples were diluted 1:5 with blank buffer, and concentrations were calculated using standard controls provided in the kit. For tissue samples, TBA and total fat were extracted with isopropanol (isopropanol volume–tissue weight = 1:10). Extraction was performed by sonication using 5-s pulses with 5-s intervals, repeated three times, followed by centrifugation at full speed at 4 °C for 20 min. The resulting supernatants were collected for subsequent analysis, and concentrations were normalized to tissue mass.

### 2.5. Gene Expression Analysis

Total RNA was isolated from mouse liver and intestine tissue. Intestinal tissue was first perfused with RNAlater (Sigma-Aldrich, Saint Louis, MO, USA, cat#R0901) for 24 h at 4 °C, and then tissues were homogenized in TRIzol reagent. RNA was extracted according to the TRIzol manufacturer’s instructions. cDNA was generated from 1 μg of DNase-treated total RNA using the iScript Reverse Transcription Supermix (Bio-Rad, Hercules, CA, USA, cat#1708841). Quantitative PCR was carried out with Advanced Universal SYBR Green Supermix (Bio-Rad, cat#725271) to measure transcript abundance. Expression levels were normalized to the housekeeping gene 36B4, and fold changes were calculated using the 2^−ΔΔCT^ method [34,35]. Primer sequences are provided in Supplementary Table S1.

### 2.6. Statistical Analysis

Genetic and environmental factors were tightly controlled in these experiments, and variability for most quantitative endpoints was expected to fall within 15–20%. Power analysis (α = 0.05, two-tailed) indicated that ~10 animals per group would provide sufficient power to detect statistically meaningful differences in primary outcomes. Accordingly, group sizes of at least 5 animals were used in individual experiments, with two independent experiments performed and combined to achieve ~10–12 animals per group in pooled analyses. Statistical analyses were performed using GraphPad Prism 9.0 (GraphPad Software, Inc., San Diego, CA, USA). For comparisons between two groups, unpaired Student’s *t*-tests were applied. For experiments involving more than two groups, one-way or two-way ANOVA was conducted, followed by Tukey’s or Dunn’s multiple comparisons test, as appropriate. The biological unit was the individual mouse. Data are presented as mean ± SEM, and statistical significance was set at *p* < 0.05 unless otherwise indicated. All experiments were designed, performed, and reported in accordance with current guidelines and best practices to ensure rigor, reproducibility, and transparency.

## 3. Results

### 3.1. Diets with Different Fat Levels Exert Distinct Effects on Body Weight and Circulating Metabolites

**Figure 1:**
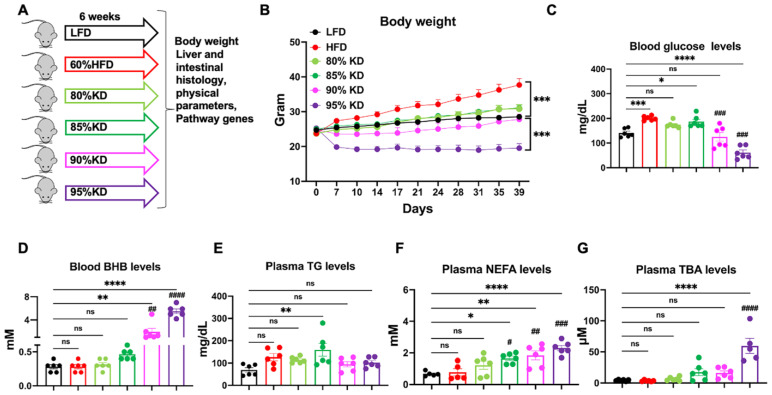
Effects of different ketogenic diets (KDs) on body weight and circulating metabolites in wild-type (WT) C57BL/6J mice. (**A**) Schematic of the dietary intervention. Eight-week-old C57BL/6J mice were fed a low-fat diet (LFD, 10% fat), a high-fat diet (HFD, 60% fat), or carbohydrate-free ketogenic diets (KD) containing 80%, 85%, 90%, or 95% fat for 6 weeks before end-point tissue harvest. (**B**) Body weight changes during the 6-week feeding period across all diet groups. (**C**) Blood glucose levels across all diet groups at end point. (**D**) Blood β-hydroxybutyrate (BHB) concentrations across all diet groups at end point. (**E**) Plasma Triglyceride (TG) levels at end point. (**F**) Plasma non-esterified fatty acid (NEFA) levels across all diet groups at end point. (**G**) Plasma bile acid (TBA) levels across all diet groups at end point. Data are presented as mean ± standard error of the mean (SEM), with n = 5–6 mice per group. Statistical analyses were performed using one-way analysis of variance (ANOVA) followed by multiple comparisons. Significance is indicated as * *p* < 0.05, ** *p* < 0.01, *** *p* < 0.001, **** *p* < 0.0001 all other diets vs. LFD; ^#^ *p* < 0.05, ^##^ *p* < 0.01, ^###^ *p* < 0.001, ^####^ *p* < 0.0001 all other diets vs. HFD; ns, not significant.

To evaluate how dietary fat content influences systemic metabolism, WT C57BL/6J mice were fed a LFD, a HFD (60% fat), or KDs containing 80%, 85%, 90%, or 95% of total calories from fat for 6 weeks (Figure 1A). Body weight progressively increased in HFD-fed mice compared with LFD-fed controls (Figure 1B). By contrast, 95% KD-fed mice showed a sustained reduction in body weight throughout the study, whereas mice on a 90% KD initially lost weight but gradually regained it, reaching levels comparable to LFD-fed mice by 6 weeks (Figure 1B). This rebound suggests an adaptive response to the 90% KD that limits further weight loss after the early phase. Unexpectedly, 80% and 85% KDs did not reduce body weight at any point; instead, by the end of the study, mice in these groups showed a mild increase relative to LFD (Figure 1B).

In parallel, blood glucose was significantly elevated in HFD-fed mice compared with LFD. Both the 90% and 95% KDs markedly reduced blood glucose, whereas the 80% and 85% KDs failed to produce meaningful improvements (Figure 1C). Circulating BHB, a hallmark of systemic ketosis, remained low in LFD, HFD, 80%, and 85% KD groups but was robustly elevated in mice fed 90% and 95% KDs (Figure 1D). This indicates that only very high-fat KDs reliably induced systemic BHB production and established a state of nutritional ketosis.

In addition, plasma lipid and BA profiles were also influenced by dietary composition. TG levels showed only modest differences among groups, with a slight reduction in the 95% KD group (Figure 1E). In contrast, NEFAs were significantly elevated in 85%, 90%, and 95% KD-fed mice compared with LFD and HFD (Figure 1F). Plasma TBA concentrations were also strongly increased in the 95% KD group (Figure 1G), consistent with enhanced lipid absorption and BA turnover under extreme fat intake. These findings suggest that triglycerides are efficiently absorbed and converted into free fatty acids (FFA) in very high-fat conditions, supporting a metabolic shift from carbohydrate reliance to fatty acid oxidation and ketone production. In addition, we did not observe a substantial increase in systemic inflammation after 6 weeks of KD feeding, as displayed by the absence of significant changes in spleen size (Figure S1A,B).

Taken together, these results demonstrate that only diets exceeding 85% of calories from fat consistently trigger systemic ketogenesis, lower blood glucose, and reduce body weight. In contrast, diets containing 80–85% fat behave more like HFD, failing to produce meaningful metabolic adaptations. This highlights > 85% dietary fat as a critical threshold for KD efficacy.

### 3.2. Hepatic Adaptations to Ketogenic Diets Reflected in Morphology, Metabolite Levels, and Gene Expression

**Figure 2:**
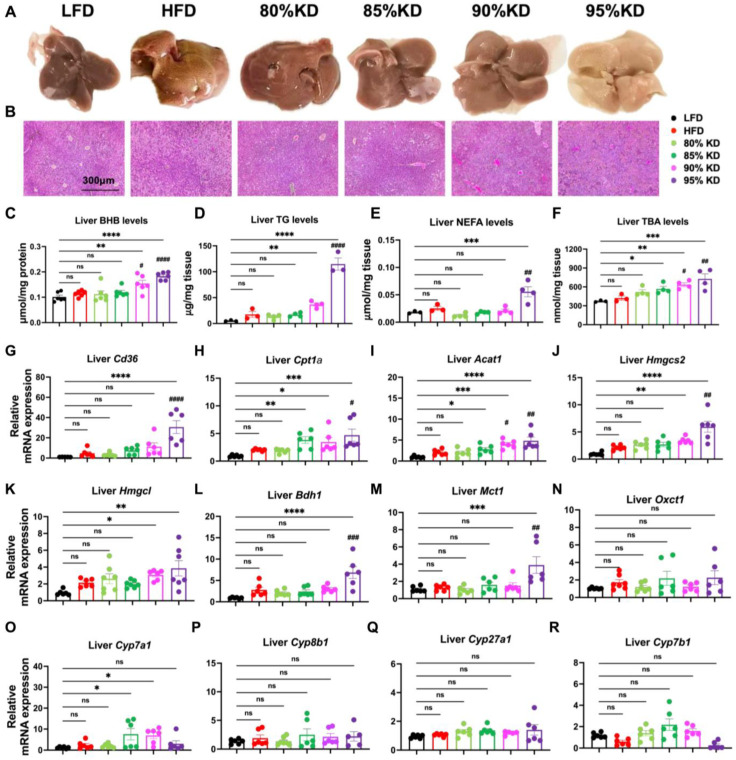
Hepatic adaptations to ketogenic diets reflected in morphology, metabolite levels, and gene expression. (**A**,**B**) Representative live images (**A**) and hematoxylin and eosin (H&E) staining (**B**) of livers from mice fed a low-fat diet (LFD, 10% fat), a high-fat diet (HFD, 60% fat), or carbohydrate-free ketogenic diets (KD) containing 80%, 85%, 90%, or 95% fat for 6 weeks. (**C**–**F**) Hepatic metabolite levels, including β-hydroxybutyrate (BHB) (**C**), triglycerides (TG) (**D**), non-esterified fatty acids (NEFA) (**E**), and total bile acids (TBA) (**F**). (**G**–**R**) Hepatic gene expression profiles measured by quantitative real-time polymerase chain reaction (qRT-PCR). Genes analyzed include those associated with fatty acid uptake and oxidation (Cd36: Cluster of Differentiation 36; Cpt1a: Carnitine Palmitoyltransferase 1A), ketogenesis and ketolysis (Acat1: Acetyl-CoA Acetyltransferase 1; Hmgcs2: 3-Hydroxy-3-Methylglutaryl-CoA Synthase 2; Hmgcl: 3-Hydroxy-3-Methylglutaryl-CoA Lyase; Bdh1: 3-Hydroxybutyrate Dehydrogenase 1; Mct1: Monocarboxylate Transporter 1; Oxct1: 3-Oxoacid CoA-Transferase 1), and bile acid synthesis (Cyp7a1: Cytochrome P450 Family 7 Subfamily A Member 1; Cyp8b1: Cytochrome P450 Family 8 Subfamily B Member 1; Cyp27a1: Cytochrome P450 Family 27 Subfamily A Member 1; Cyp7b1: Cytochrome P450 Family 7 Subfamily B Member 1). Data are presented as mean ± SEM. Statistical analyses were performed using one-way ANOVA followed by multiple comparisons. * *p* < 0.05, ** *p* < 0.01, *** *p* < 0.001, **** *p* < 0.0001 all other diets vs. LFD; ^#^ *p* < 0.05, ^##^ *p* < 0.01, ^###^ *p* < 0.001, ^####^ *p* < 0.0001 all other diets vs. HFD; ns, not significant.

In order to examine how ketogenic diets impact liver morphology and function in more detail, we compared the appearance of livers across diet groups. Livers from HFD- and 95% KD-fed mice appeared larger and paler, whereas livers from mice on moderate KDs (80% and 85%) as well as 90% KD were smaller and showed a similar color to those from LFD-fed mice (Figure 2A). Consistently, the relative liver weight (liver-to-body weight ratio) was significantly increased in 95% KD-fed mice, while no significant differences were detected in the LFD, HFD, 80%, 85%, or 90% groups after 6 weeks of feeding (Figure S1A). These observations suggest that six weeks of HFD feeding is not sufficient to induce fatty liver in this model, whereas prolonged feeding with 95% KD may already trigger liver enlargement and potential early liver injury. However, we did not observe severe systemic inflammation, as indicated by unchanged spleen weight and size (Figure S1A,B).

Histological staining further supported these observations. H&E staining revealed prominent lipid accumulation and vacuolation in livers from HFD-fed mice, whereas hepatocytes from 95% KD-fed mice showed more extensive vacuolation and disrupted architecture compared with LFD controls (Figure 2B). In contrast, the moderate-KD groups (80% and 85%) displayed relatively preserved morphology, similar to LFD-fed mice (Figure 2B). This pattern suggests that HFD promotes fatty liver progression, whereas extreme fat intake, as in the 95% KD, imposes a greater burden on the liver with increased relative liver weight to whole-body weight (Figure S1A). These findings further indicate that only moderate-fat KDs (80% and 85%) may protect the liver from diet-induced lipid deposition.

To link these phenotypic observations with liver metabolism, we next measured hepatic metabolites. BHB levels were significantly increased in the 90% and 95% KD groups, consistent with active hepatic ketogenesis (Figure 2C). In contrast, hepatic triglyceride levels showed a trend of gradual increase with higher dietary fat, with a marked induction in 90% and 95% KD-fed animals (Figure 2D). Similarly, FFAs were significantly elevated in the 95% KD group compared with LFD and HFD (Figure 2E), indicating enhanced fatty acid mobilization and oxidation. Additionally, hepatic TBA levels were significantly higher in the 85%, 90%, and 95% KD groups (Figure 2F), suggesting increased BA turnover under conditions of very high fat intake.

To further explore the molecular basis of these metabolic changes, we examined hepatic gene expression. Fatty acid uptake and oxidation genes, including Cd36 and Cpt1a, were strongly induced by 90% and 95% KDs (Figure 2G,H). Likewise, key enzymes involved in ketogenesis (Acat1, Hmgcs2, Hmgcl, and Bdh1) were significantly upregulated (Figure 2I–L). Of note, the ketone body transport-related gene Mct1 showed higher expression (Figure 2M), whereas the utilization-related gene Oxct1 (Figure 2N) exhibited little change. This suggests that hepatic ketone bodies are largely exported as an energy source rather than being extensively consumed in the liver, consistent with previous reports. By contrast, among genes regulating BA synthesis, only Cyp7a1 was significantly increased in the 85% and 90% KD groups, while Cyp8b1, Cyp27a1, and Cyp7b1 were not altered (Figure 2O–R). Taken together, these results demonstrate that KDs exceeding 85% fat remodel liver metabolism by enhancing fatty acid uptake and oxidation and promoting ketone body synthesis.

### 3.3. Intestinal Responses to Ketogenic Diets Reflected in Morphology, Metabolite Levels, and Gene Expression

**Figure 3:**
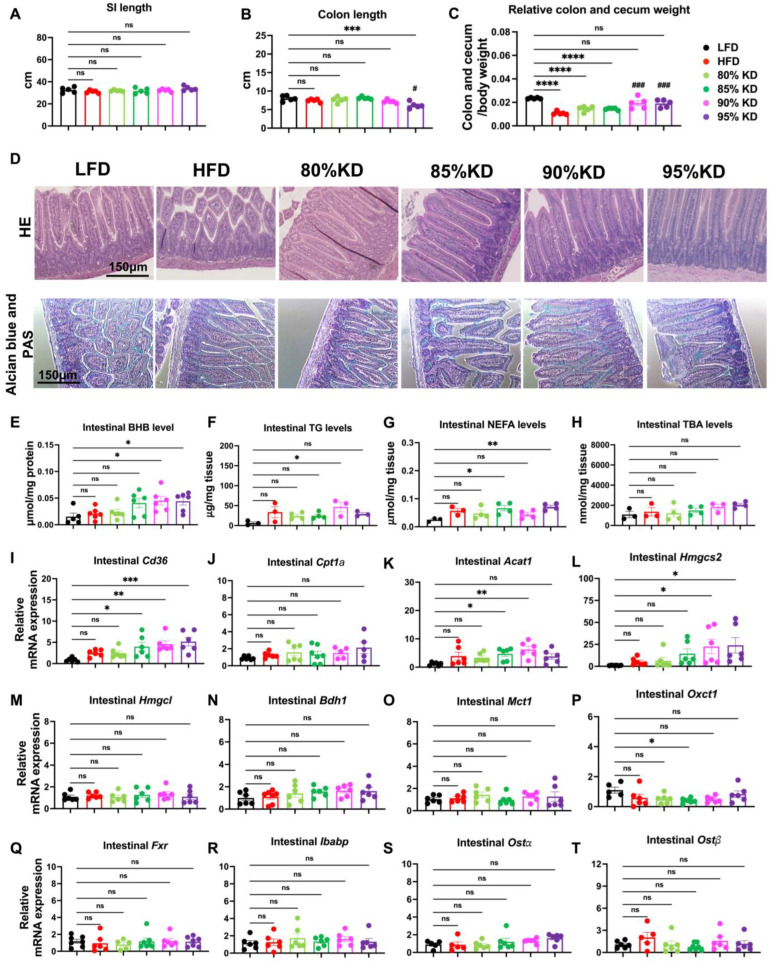
Intestinal responses to ketogenic diets reflected in morphology, metabolite levels, and gene expression. (**A**–**C**) Relative small intestine (SI) weight (**A**), colon length (**B**), and relative colon plus cecum weight (**C**) after 6 weeks of feeding with a low-fat diet (LFD, 10% fat), a high-fat diet (HFD, 60% fat), or carbohydrate-free ketogenic diets (KD) containing 80%, 85%, 90%, or 95% fat. (**D**) Representative hematoxylin and eosin (H&E) staining and Alcian Blue/periodic acid–Schiff (PAS) staining of small intestine sections across diet groups. (**E**–**H**) Intestinal metabolite levels, including β-hydroxybutyrate (BHB) (**E**), triglycerides (TG) (**F**), non-esterified fatty acids (NEFA) (**G**), and total bile acids (TBA) (**H**). (**I**–**T**) Relative intestinal gene expression profiles measured by quantitative polymerase chain reaction (qPCR). Genes analyzed include fatty acid uptake/oxidation (Cd36: Cluster of Differentiation 36; Cpt1a: Carnitine Palmitoyltransferase 1A) (**I**,**J**); ketogenesis (Acat1: Acetyl-CoA Acetyltransferase 1; Hmgcs2: 3-Hydroxy-3-Methylglutaryl-CoA Synthase 2; Hmgcl: 3-Hydroxy-3-Methylglutaryl-CoA Lyase; Bdh1: 3-Hydroxybutyrate Dehydrogenase 1) (**K**–**N**); ketone body transport and utilization (Mct1: Monocarboxylate Transporter 1; Oxct1: 3-Oxoacid CoA-Transferase 1) (**O**,**P**); and bile acid signaling/transport (Fxr: Farnesoid X Receptor; Ibabp: Ileal Bile Acid-Binding Protein; Ostα: Organic Solute Transporter Alpha; Ostβ: Organic Solute Transporter Beta) (**Q**–**T**). Data are presented as mean ± standard error of the mean (SEM). Statistical analyses were performed using one-way analysis of variance (ANOVA) with multiple comparisons. Significance is indicated as * *p* < 0.05, ** *p* < 0.01, *** *p* < 0.001, **** *p* < 0.0001 all other diets vs. LFD; ^#^ *p* < 0.05, ^###^ *p* < 0.001 all other diets vs. HFD; ns, not significant.

To further investigate intestinal responses to ketogenic diets, we examined morphological changes in both the small intestine and colon. Small intestine (SI) length did not differ substantially across diet groups (Figure 3A and Figure S1C,D). By contrast, colon length was shortened only in mice fed a 95% KD (Figure 3B and Figure S1E). Meanwhile, the relative colon plus cecum weight was significantly decreased in HFD-, 80% KD-, and 85% KD-fed mice, whereas no significant changes were observed in the 90% or 95% KD groups compared with LFD (Figure 3C).

Histological analysis confirmed diet-dependent structural changes as well. H&E staining revealed more compact villus structures in the 90% and 95% KD groups, whereas LFD and moderate-KD groups displayed preserved intestinal morphology (Figure 3D), suggesting a potential adaption to increased fat intake. While Alcian Blue/PAS staining did not show significant changes across different groups (Figure 3D).

We next assessed intestinal metabolites. Intestinal BHB levels were significantly increased in the 90% and 95% KD groups compared with ND, consistent with enhanced local ketone production (Figure 3E). In contrast, intestinal TG and FFA levels showed modest but consistent increases in both HFD- and KD-fed mice (Figure 3F,G), suggesting greater intestinal uptake of dietary lipids and an increased availability of FFAs as substrates for β-oxidation and ketogenesis. By comparison, intestinal BA levels remained largely unchanged across all diet groups (Figure 3H).

To connect these changes to molecular regulation, we also examined intestinal gene expression. Fatty acid uptake gene (Cd36) was significantly upregulated in the 90% and 95% KD groups (Figure 3I), whereas the expression of Cpt1a, a key fatty acid oxidation gene, remained unchanged (Figure 3J). Similarly, ketogenic enzymes (Acat1, Hmgcs2) were elevated under high-KD feeding (Figure 3K,L), although other ketogenic genes (Hmgcl, Bdh1) showed no significant differences (Figure 3M,N). The ketone transporter Mct1 and utilization gene Oxct1 remained unchanged across diets (Figure 3O,P). This suggests that intestinal ketone bodies may serve primarily local functions, such as mediating intracellular signaling or supporting energy metabolism in enterocytes. With respect to BA signaling, Fxr and its target Ibabp were not significantly altered across diet groups (Figure 3Q,R). Similarly, the basolateral BA transporters Ostα and Ostβ showed no significant differences (Figure 3S,T), consistent with the unchanged intestinal BA levels (Figure 3H).

In addition, an 80% fat, 5% carbohydrate diet did not significantly alter systemic, hepatic, or intestinal parameters. Circulating BHB, TG, NEFA, and BA levels remained unchanged, compared with ND (Figure S2A–G). Relative liver weight SI length as well as the metabolites including TGs, FFAs, and BA levels were also unaffected (Figure S2H–T). These results confirm that an 80% KD fails to robustly induce ketogenesis or metabolic remodeling systematically.

Taken together, these data indicate that only very high KDs (>90%) remodel intestinal morphology and induce local ketogenesis, whereas moderate KDs (80–85%) exert minimal effects. The elevated intestinal BHB levels and upregulation of ketogenic enzymes highlight a potential role for the gut epithelium in contributing to the local ketone pool, which may influence enterocyte energy metabolism or paracrine signaling within the intestine.

### 3.4. Effects of Ketogenic Diet Interventions on Diet-Induced Obesity on Systemic Metabolic Parameters in Mice

**Figure 4:**
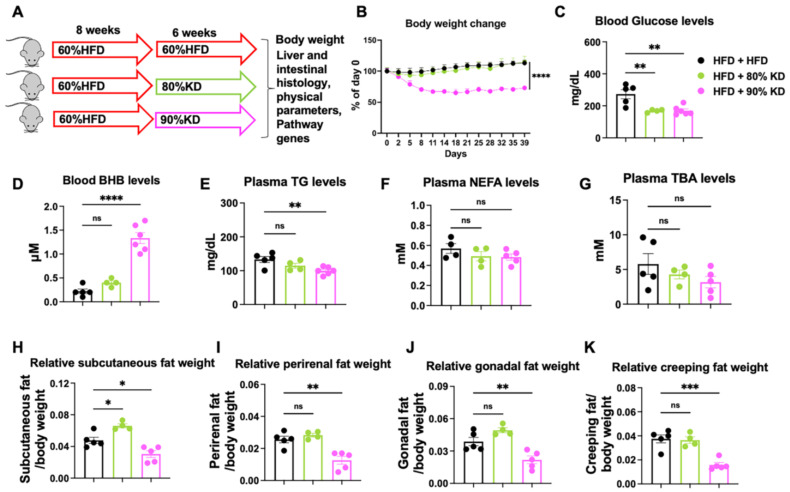
Effects of ketogenic diet interventions on diet-induced obesity on systemic metabolic parameters in mice. (**A**) Schematic of the experimental design. C57BL/6J mice were pre-fed a high-fat diet (HFD, 60% fat) for 8 weeks to induce obesity and then maintained on HFD or switched to carbohydrate-free 80% KD or 90% KD for 6 weeks. During this period, body weights were monitored, and tissues and blood samples were collected for histological analysis, physiological measurements, and metabolic gene expression profiling. (**B**) Body weight changes during the 6-week intervention across the three groups. (**C**) Blood glucose concentrations at end point. (**D**) Circulating β-hydroxybutyrate (BHB) levels at end point. (**E**) Plasma triglyceride (TG) levels at end point. (**F**) Plasma non-esterified fatty acid (NEFA) levels at end point. (**G**) Plasma total bile acid (TBA) levels at end point. (**H**–**K**) Relative fat depot weights normalized to body weight, including subcutaneous fat (**H**), perirenal fat (**I**), gonadal fat (**J**), and creeping fat (**K**). Data are presented as mean ± standard error of the mean (SEM). Statistical analyses were performed using one-way analysis of variance (ANOVA) with multiple comparisons. Significance is indicated as * *p* < 0.05, ** *p* < 0.01, *** *p* < 0.001, **** *p* < 0.0001, ns, not significant; treatment groups vs. HFD group.

To investigate which ketogenic diets could help reduce weight, we examined the systemic metabolic effects of ketogenic diet interventions in the setting of diet-induced obesity. Mice were pre-fed a 60% HFD for 8 weeks to induce obesity (DIO) and then treated with 80% or 90% KD for 6 weeks (Figure 4A). In contrast to 80% KD, only mice on 90% KD showed a progressive reduction in body weight compared to HFD-fed controls (Figure 4B). Fasting blood glucose was significantly decreased in both 80% and 90% KD-fed groups, with the lowest levels observed in the 90% KD group (Figure 4C). Circulating BHB levels were markedly elevated only in 90% KD-fed mice, confirming induction of systemic ketogenesis (Figure 4D). Plasma lipid and TBA profiles were next assessed. Triglyceride concentrations were modestly reduced by 90% KD, whereas NEFAs and TBA levels showed a decreasing trend but were not significantly altered among groups (Figure 4E–G). Analysis of adipose tissue distribution revealed depot-specific effects: subcutaneous fat was significantly increased in 80% KD, but markedly decreased in 90% KD-fed mice, whereas perirenal, gonadal, and creeping fat were reduced primarily in the 90% KD group (Figure 4H–K and Figure S3A). Together, these results indicate that high-level KD feeding reduces body weight, improves glycemia, and remodels adipose tissue distribution without broadly altering systemic lipid or TBA levels.

In addition, we treated DIO mice with an 80% fat, 5% carbohydrate KD for 6 weeks. This regimen did not change body weight but significantly reduced fasting glucose compared with HFD controls, while circulating BHB, TG, and NEFA remained unchanged (Figure S4A–F). Plasma TBAs were decreased (Figure S4G). Interestingly, subcutaneous and gonadal fat depots were even increased in KD-fed mice, whereas perirenal and creeping fat were unaffected (Figure S4H–K). These results suggest that this KD scheme may improve glucose sensitivity but is not effective for overall weight loss.

### 3.5. Effects of Ketogenic Diet Interventions on Hepatic Morphology, Lipid Accumulation, and Gene Expression

**Figure 5:**
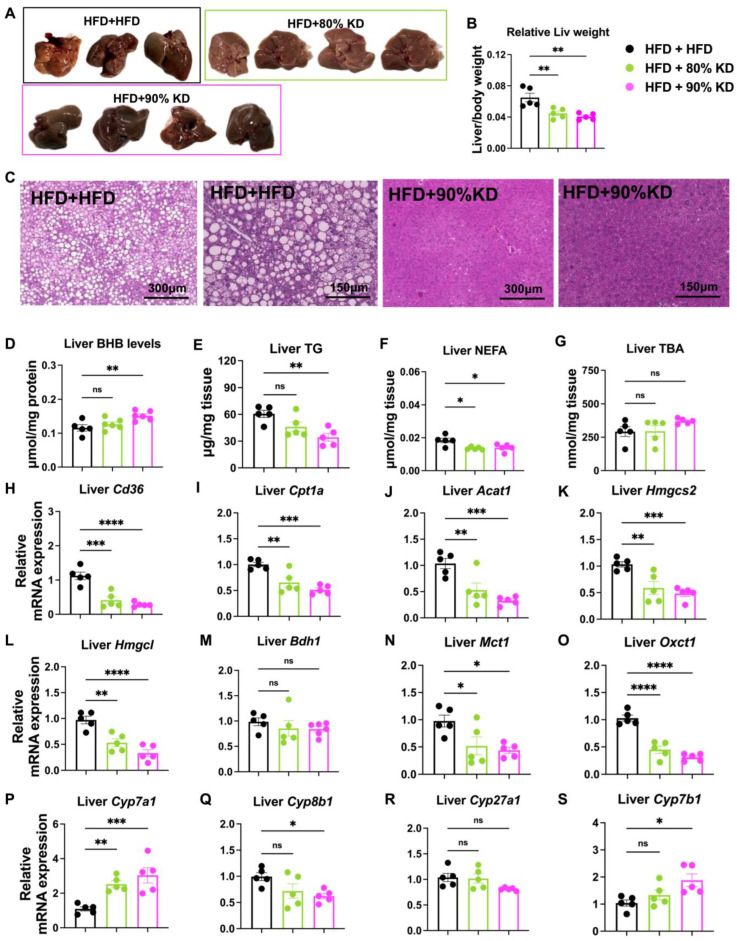
Effects of ketogenic diet interventions on hepatic morphology, lipid accumulation, and gene expression. (**A**) Representative gross liver images from mice fed a high-fat diet (HFD, 60% fat), 80% KD, or 90% KD for 6 weeks after obesity induction. (**B**) Relative liver weight normalized to body weight in the three groups. (**C**) Representative hematoxylin and eosin (H&E)–stained liver sections at 10× and 20× magnification from HFD- and 90% KD–fed mice. (**D**–**G**) Hepatic metabolite levels, including β-hydroxybutyrate (BHB) (**D**), triglycerides (TG) (**E**), non-esterified fatty acids (NEFA) (**F**), and total bile acids (TBA) (**G**). (**H**–**S**) Hepatic gene expression profiles measured by quantitative real-time polymerase chain reaction (qRT-PCR). Genes analyzed include those associated with fatty acid uptake and oxidation (Cd36: Cluster of Differentiation 36; Cpt1a: Carnitine Palmitoyltransferase 1A), ketogenesis and ketolysis (Acat1: Acetyl-CoA Acetyltransferase 1; Hmgcs2: 3-Hydroxy-3-Methylglutaryl-CoA Synthase 2; Hmgcl: 3-Hydroxy-3-Methylglutaryl-CoA Lyase; Bdh1: 3-Hydroxybutyrate Dehydrogenase 1; Mct1: Monocarboxylate Transporter 1; Oxct1: 3-Oxoacid CoA-Transferase 1), and bile acid synthesis (Cyp7a1: Cytochrome P450 Family 7 Subfamily A Member 1; Cyp8b1: Cytochrome P450 Family 8 Subfamily B Member 1; Cyp27a1: Cytochrome P450 Family 27 Subfamily A Member 1; Cyp7b1: Cytochrome P450 Family 7 Subfamily B Member 1). Data are presented as mean ± standard error of the mean (SEM). Statistical analyses were performed using one-way analysis of variance (ANOVA) with multiple comparisons. Significance is indicated as * *p* < 0.05, ** *p* < 0.01, *** *p* < 0.001, **** *p* < 0.0001; ns, not significant; treatment groups vs. HFD group.

To examine the hepatic response to ketogenic diet interventions in diet-induced obesity, we compared liver morphology, weight, metabolite levels, and transcriptional programs. Gross liver appearance and relative liver weight showed hepatomegaly in HFD-fed mice, which was significantly reduced by 90% KD, while 80% KD showed no effect (Figure 5A,B and Figure S3B). Histological analysis further revealed extensive lipid droplet accumulation under HFD, whereas 90% KD feeding markedly reduced hepatic steatosis (Figure 5C).

Biochemical measurements demonstrated that hepatic BHB concentrations were significantly increased in 90% KD-fed mice, confirming induction of ketogenesis (Figure 5D). In contrast, hepatic triglyceride and NEFA levels showed a significant reduction in both KD groups compared to HFD (Figure 5E,F). Given the reduced liver size and histological evidence of less steatosis (Figure 5A–C and Figure S3B), this trend likely reflects a true reduction in hepatic lipid burden that was not fully captured at the biochemical level within six weeks. Mobilized fatty acids from adipose depots may be preferentially oxidized in peripheral tissues at the 6-week time point, while the liver has already reached a relatively steady TG and FFA pool through balanced influx and oxidation. Similarly, hepatic TBA levels trended downward but remained unchanged, consistent with tight homeostatic regulation and suggesting reduced steatosis with preserved liver function (Figure 5G).

Expression analysis first revealed that the fatty acid uptake gene Cd36 and the β-oxidation gene Cpt1a were significantly reduced in 80% and 90% KD-fed livers compared with HFD controls (Figure 5H,I). This downregulation is surprising given the substantial body weight loss observed under KD but likely reflects a state of metabolic adaptation in which mobilized fatty acids are preferentially oxidized in peripheral tissues such as muscle, heart and brain at the 6-week time point, reducing the requirement for hepatic lipid uptake. In line with this, transcripts for ketogenesis and ketolysis enzymes, including Acat1, Hmgcs2, Hmgcl, and Mct1, were also downregulated (Figure 5J–L,N), while Bdh1 was unchanged (Figure 5M) and the ketone utilization gene Oxct1 was markedly reduced (Figure 5O). Nevertheless, circulating ketone levels remained robustly elevated in 90% KD-fed mice (Figure 4D), suggesting that ketone production is sustained primarily through substrate availability or post-translational control rather than persistent induction of mRNA expression. Genes involved in BA synthesis showed mixed regulation, with Cyp7a1 significantly increased, whereas Cyp8b1 and Cyp7b1 showed a remarkable reduction (Figure 5P–S). Together, these findings highlight a paradoxical metabolic state under prolonged KD feeding, in which systemic ketosis and weight loss occur even as hepatic gene programs for fatty acid uptake, oxidation, and ketone metabolism are suppressed—likely reflecting metabolic adaptation, substrate-driven flux, and contributions from extrahepatic tissues.

### 3.6. Effects of Ketogenic Diet Interventions on Intestinal Morphology, Lipid Accumulation, and Gene Expression

**Figure 6:**
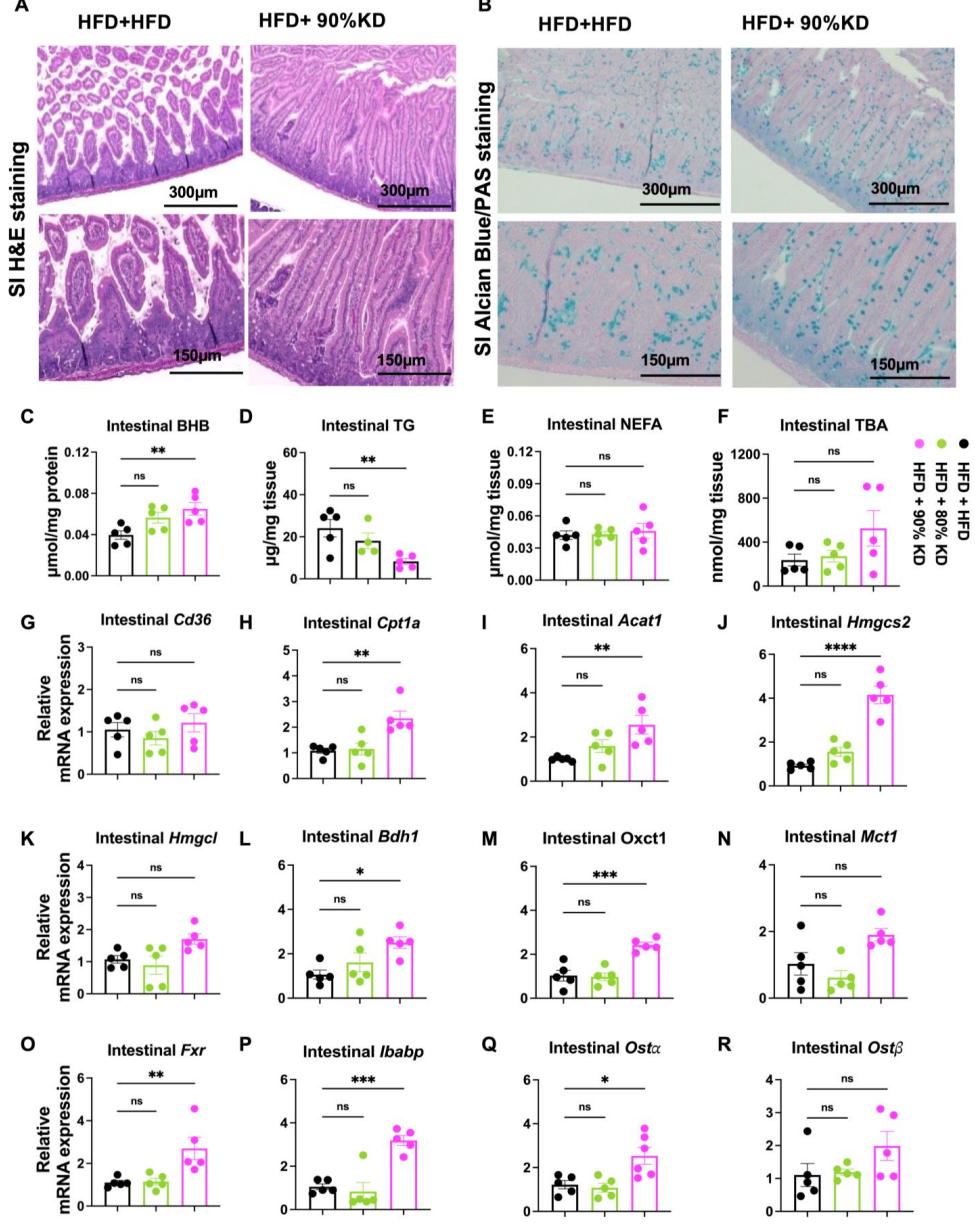
Effects of ketogenic diet interventions on intestinal morphology, lipid accumulation, and gene expression. (**A**,**B**) Representative hematoxylin and eosin (H&E) (**A**) and Alcian Blue/periodic acid–Schiff (PAS) (**B**)–stained sections of small intestine from mice fed high-fat diet (HFD, 60% fat), HFD + 80% KD, or HFD + 90% KD, shown at 300 μm and 150 μm magnification. (**C**–**F**) Intestinal metabolite levels, including β-hydroxybutyrate (BHB) (**C**), triglycerides (TG) (**D**), non-esterified fatty acids (NEFA) (**E**), and total bile acids (TBA) (**F**). (**G**–**R**) Relative intestinal mRNA expression profiles measured by quantitative real-time polymerase chain reaction (qRT-PCR). Genes analyzed include those related to fatty acid uptake and oxidation (Cd36: Cluster of Differentiation 36; Cpt1a: Carnitine Palmitoyltransferase 1A), ketogenesis and ketolysis (Acat1: Acetyl-CoA Acetyltransferase 1; Hmgcs2: 3-Hydroxy-3-Methylglutaryl-CoA Synthase 2; Hmgcl: 3-Hydroxy-3-Methylglutaryl-CoA Lyase; Bdh1: 3-Hydroxybutyrate Dehydrogenase 1; Oxct1: 3-Oxoacid CoA-Transferase 1; Mct1: Monocarboxylate Transporter 1), and bile acid signaling and transport (Fxr: Farnesoid X Receptor; Ibabp: Ileal Bile Acid-Binding Protein; Ostα: Organic Solute Transporter Alpha; Ostβ: Organic Solute Transporter Beta). Data are presented as mean ± standard error of the mean (SEM). Statistical analyses were performed using one-way analysis of variance (ANOVA) with multiple comparisons. Significance is indicated as * *p* < 0.05, ** *p* < 0.01, *** *p* < 0.001, **** *p* < 0.0001, ns, not significant; treatment groups vs. HFD group.

To evaluate the intestinal response to ketogenic diet interventions, we assessed morphology, barrier function, metabolites, and transcriptional programs. Since only the 90% KD group showed dramatic systemic changes, we next examined its effects on intestinal structure and function. Both 80% and 90% KDs did not alter the length of the small intestine or colon (Figure S3C,D). Spleen weight was slightly reduced in the 80% KD group but remained unchanged in the 90% KD group (Figure S3E), indicating no systemic inflammation. H&E staining revealed that HFD feeding damaged villus length in the small intestine, whereas 90% KD successfully preserved villus morphology (Figure 6A). Alcian Blue/PAS staining further indicated that 90% KD increased Paneth cell numbers in the crypt and enhanced goblet cell abundance along the villi (Figure 6B). These changes may reflect a protective effect of 90% KD on the epithelial stem-cell niche and secretory lineage differentiation, promoting mucosal defense and barrier stability.

Consistent with improved mucosal integrity, intestinal permeability measured by fluorescein isothiocyanate (FITC)-dextran was significantly reduced in 90% KD-fed mice compared with HFD controls (Figure S3F). Biochemical analysis showed that intestinal BHB levels were increased under KD, with a significant elevation in the 90% KD group (Figure 6C). In contrast, intestinal TG levels displayed a significant reduction, suggesting reduced TG absorption after 6 weeks of KD adaptation (Figure 6D). Both intestinal NEFA levels and TBA levels remained unchanged across groups (Figure 6E,F).

Gene expression profiling revealed selective transcriptional remodeling under KD. Intestinal Cd36, a gene responsible for fatty acid uptake, was not significantly altered (Figure 6G), indicating that lipid absorption capacity remained stable. Despite this, intestinal TG levels were reduced, suggesting changes in downstream lipid handling. Consistently, Cpt1a, a key fatty acid oxidation gene, was significantly upregulated (Figure 6H), pointing to enhanced lipid oxidation. Among the ketogenesis genes, Acat1, Hmgcs2, and Bdh1 were significantly increased in intestines of 90% KD–fed mice (Figure 6I,J,L), whereas Hmgcl was unchanged (Figure 6K). The ketone utilization gene Oxct1 was also significantly upregulated (Figure 6M), while Mct1 expression remained unaltered (Figure 6N). Additionally, Fxr and it downstream genes like Ibabp, Ostα and Ostβ increased in intestines of 90% KD–fed mice (Figure 6O–R). These findings suggest that the intestine ramps up ketone production and simultaneously enhances its ability to utilize ketones, but without increasing export. It suggests that intestinal ketogenesis operates as a self-sustained program to meet local energy demands under low-glucose conditions. In contrast, the liver downregulates ketogenic genes after chronic KD, maintaining systemic ketone output via substrate flux or post-translational control. Thus, whereas the liver supports whole-body energy supply, the intestine engages ketogenesis as a cell-autonomous response.

## 4. Discussion

In this study, we systematically examined the effects of KDs of varying fat content both in WT mice as a direct feeding intervention and in DIO mice as a treatment strategy. Our findings highlight that both diet composition and host metabolic status determine the systemic and tissue-specific outcomes of KD feeding.

Feeding WT mice with diets ranging from chow to extreme high-fat ketogenic formulations revealed a clear threshold effect: only diets with ≥90% fat reliably lowered glucose, reduced weight, and induced nutritional ketosis, whereas 80 and 85% KDs, despite being carbohydrate-free, failed to elevate ketones or prevent weight gain. This reflects species-specific features of murine metabolism, which favors glucose and resists ketosis unless fat intake is extremely high [36]. The body weight reduction observed in the 95% KD group may be partly attributable to protein insufficiency, as this diet contains substantially less protein than other KD formulations. Supporting this notion, previous studies have demonstrated that very low-protein diets (1–5 kcal%) can reduce body weight even in the context of high fat intake (60 kcal%) [37] The hepatic response paralleled this systemic threshold. While HFD promoted lipid accumulation, only the 95% KD group developed hepatomegaly, vacuolation, and elevated TG and NEFA levels, consistent with early liver injury. In contrast, the 90% KDs preserved hepatic morphology while still supporting ketone synthesis. Gene expression profiling revealed induction of fatty acid uptake and oxidation genes (Cd36, Cpt1a) and key ketogenic enzymes (Acat1, Hmgcs2, Hmgcl, Bdh1) in both 90% and 95% KDs, consistent with elevated hepatic BHB. Importantly, Oxct1 expression remained low, whereas Mct1 was upregulated, reinforcing the liver’s role as a major producer and exporter of ketone bodies. These findings suggest that while the liver is essential for establishing systemic ketosis under very high-fat feeding, extreme fat intake (95% KD) may also impose metabolic stress that predisposes to liver injury.

The intestine also showed marked responses to very high-fat KDs. Relative SI weight increased in 90% and 95% KD-fed mice, and villus morphology became more compact, with altered mucus production at the highest fat levels. Intestinal BHB levels were elevated in these groups, accompanied by strong induction of Acat1 and Hmgcs2. Given the intestine’s low basal Hmgcs2 expression, this induction appears particularly striking compared with the liver. By contrast, Oxct1 was not significantly upregulated, suggesting that enterocyte-derived ketones may serve functions beyond oxidative fuel. Unlike the liver, the intestine’s ketogenic program appears tailored for local functions, such as energy support during carbohydrate restriction and regulation of epithelial renewal, barrier defense, and immune signaling [18,26,33]. Thus, while the liver governs systemic ketone supply, the intestine may exploit a diet-inducible ketogenic program to regulate local metabolism.

In obese mice maintained on HFD, therapeutic outcomes depended strongly on diet composition. The 80% KD lowered fasting glucose and reduced hepatomegaly, but did not reduce weight or raise systemic ketones, and even increased some adipose depots, indicating improved glycemic sensitivity without whole-body reprogramming. In contrast, the 90% KD induced robust weight loss, sustained ketosis, and improved both hepatic and intestinal phenotypes, demonstrating that only stricter formulations effectively reverse obesity-related disturbances. At the hepatic level, 90% KD feeding reduced hepatomegaly despite only modest changes in hepatic TG and NEFA content. This pattern suggests that the liver establishes a new steady-state balance between lipid influx, oxidation, and export rather than simply depleting its lipid stores. Interestingly, hepatic expression of fatty acid uptake, oxidation, and ketogenesis genes was not elevated, reinforcing the concept that long-term hepatic ketone production is maintained primarily through substrate-driven flux and post-translational regulation rather than persistent transcriptional induction. These features highlight the liver’s role as a central coordinator of systemic ketosis, providing ketone bodies to peripheral tissues while avoiding excessive lipid accumulation.

In contrast to the liver, the intestine displayed a distinct transcriptional and functional profile. KD preserved villus length and improved barrier integrity, as evidenced by reduced FITC–dextran permeability. Intestinal BHB levels were elevated under 90% KD, accompanied by upregulation of Acat1, Hmgcs2, Bdh1 and, notably, Oxct1. Unlike in WT mice (not DIO mice) where Oxct1 remained unchanged, its induction in obese mice suggests that enterocytes not only generate but also actively utilize ketone bodies as an energy source under therapeutic KD feeding. This highlights a self-contained ketogenesis/ketolysis system within the intestinal epithelium that may sustain local energy metabolism during carbohydrate restriction. Unlike the liver, which coordinates systemic energy supply, the intestine appears to rely on ketone production for cell-autonomous adaptation, potentially shaped by local cues such as bile acid signaling, microbiota-derived metabolites, and crypt–villus gradients. The intestine showed a distinct pro-ketolysis system, which highlights the intestine as an active metabolic site supporting local energy demands and barrier protection [18,26,33], in contrast to the liver’s systemic role.

Taken together, our results reveal that ketogenic diets exert distinct, context-dependent effects in healthy versus obese mice. First, in WT animals, 80% KD failed to induce ketosis and even promoted weight gain, whereas in obese mice the same diet, while not reducing weight, improved fasting glucose, suggesting that glucose handling is more diet-sensitive in obesity, while lean animals remain resistant to metabolic switching and prone to fat storage. Second, in WT mice, six weeks of 90% KD sustained strong hepatic transcriptional induction of ketogenesis with elevated TGs, NEFAs, and BAs, reflecting reliance on the liver for systemic ketone production. In obese mice, however, 90% KD drove weight loss and systemic ketosis with reduced hepatic lipid burden but increased BAs, indicating peripheral tissues act as sinks for fatty acids and ketones. Third, both WT and obese mice showed robust intestinal Hmgcs2 induction under 90% KD, but only obese mice upregulated Oxct1, pointing to active intestinal ketone utilization. This suggests intestinal ketogenesis functions mainly in signaling in lean animals but provides direct energy support in the obese state.

These findings carry important translational implications for KDs in humans. Unlike mice, which are more carbohydrate dependent and resistant to ketosis, humans typically achieve ketosis at lower fat thresholds [38,39]. However, individual variability shaped by prior diet, metabolic state, and genetics means circulating ketone levels remain the most reliable biomarker for tailoring ketogenic interventions [40]. While KDs can provide metabolic benefits, long-term adherence has been linked to potential cardiac risks [41,42], highlighting the importance of careful monitoring. Another important question is duration and scheduling. Our study examined continuous feeding, but intermittent or cyclic regimens in humans may yield similar benefits with fewer risks. Finally, although exogenous BHB supplementation can mimic ketosis [43], intestinal ketogenesis may have unique functions in epithelial renewal, mucosal defense, and host microbiota interactions, which warrants further investigation. Overall, these considerations emphasize the need for personalized and flexible strategies when translating KDs into human obesity treatment.

## 5. Conclusions

In conclusion, our study identifies > 90% dietary fat as a critical threshold for inducing robust ketosis in mice and highlights distinct roles of the liver and intestine in systemic versus local ketone metabolism. The findings emphasize that both host metabolic state and diet composition determine the outcomes of KD feeding, with obesity favoring peripheral utilization and intestinal ketone consumption.

## Figures and Tables

**Table 1:** Nutrient composition of diets (protein, carbohydrates, and fat).

Diet	LFD		HFD		80% KD		80% Fat, 5% Carb KD		85% KD		90% KD		95% KD	
	g%	kcal%	g%	kcal%	g%	kcal%	g%	kcal%	g%	kcal%	g%	kcal%	g%	kcal%
Protein	19	20	26	20	31	20	23	15	24	15	17	10	9	5
Carbohydrate	67	70	26	20	0	0	8	5	0	0	0	0	0	0
Fat	4	10	35	60	54	80	55	80	60	85	67	90	74	95
Total		100		100		100		100		100		100		100
kcal/g	3.9		5.2		6.1		6.1		6.4		6.7		7.0	

## Data Availability

The original contributions presented in this study are included in the article/Supplementary Material. Further inquiries can be directed to the corresponding author.

## Supplementary Materials

The following supporting information can be downloaded at: https://www.mdpi.com/article/10.3390/nu17203203/s1, Figure S1: Metabolic adaptations to different ketogenic diets. Figure S2. Minimal effects of an 80% fat, 5% carbohydrate ketogenic diet on systemic, hepatic, and intestinal parameters. Figure S3. Live images of liver, different fat depots, small intestine and colon in DIO mice under 80% KD and 90% KD treatment. Figure S4. Effects of 80% KD on systemic metabolic parameters, adipose tissue distribution, and organ morphology in diet-induced obese mice. Table S1. Diet information (related to Table 1). Table S2. Experimental flowchart showing the correspondence between figures and experimental schemes. Table S3. Primer sequences.

## Abbreviations

The following abbreviations are used in this manuscript:

AAALAC	Association for Assessment and Accreditation of Laboratory Animal Care
Acat1	Acetyl-CoA Acetyltransferase 1
BA	Bile acid
Bdh1	3-Hydroxybutyrate Dehydrogenase 1
BHB	Beta-hydroxybutyrate
DIO	Diet-induced obese
Cd36	Cluster of Differentiation 36
Cpt1a	Carnitine Palmitoyltransferase 1A
Cyp7a1	Cytochrome P450 family 7 subfamily A member 1
Cyp7b1	Cytochrome P450 family 7 subfamily B member 1
Cyp8b1	Cytochrome P450 family 8 subfamily B member 1
Cyp27a1	Cytochrome P450 family 27 subfamily A member 1
FFA	Free fatty acid
FITC	Fluorescein Isothiocyanate
Fxr	Farnesoid X Receptor
HDACs	Histone deacetylases
H&E	Hematoxylin & Eosin
HFD	High-fat diet
Hmgcs2	3-Hydroxy-3-Methylglutaryl-CoA Synthase 2
Hmgcl	3-Hydroxy-3-Methylglutaryl-CoA Lyase
ISC	Intestinal Stem Cells
IACUC	Institutional Animal Care and Use Committee
Ibabp	Ileal Bile Acid-Binding Protein
KD	Ketogenic diet
Mct1	Monocarboxylate transporter 1
ND	Normal diet
NEFA	Non-esterified fatty acids
Ostα	Organic solute transporter alpha
Ostβ	Organic solute transporter beta
Oxct1	3-oxoacid CoA-transferase 1
SI	small intestine
TBA	Total bile acid
TDCA	Taurodeoxycholic acid
TG	Triglyceride
TUDCA	Tauroursodeoxycholic acid
Th17	T helper 17 cells
WT	Wild type

## References

[1] Bluher M. (2019). Obesity: Global epidemiology and pathogenesis. Nat. Rev. Endocrinol..

[2] Heymsfield S.B., Wadden T.A. (2017). Mechanisms, Pathophysiology, and Management of Obesity. N. Engl. J. Med..

[3] Adolph T.E., Tilg H. (2024). Western diets and chronic diseases. Nat. Med..

[4] Rakhra V., Galappaththy S.L., Bulchandani S., Cabandugama P.K. (2020). Obesity and the Western Diet: How We Got Here. Mo. Med..

[5] de Moura E.D.M., Dos Reis S.A., da Conceicao L.L., Sediyama C., Pereira S.S., de Oliveira L.L., Gouveia Peluzio M.D.C., Martinez J.A., Milagro F.I. (2021). Diet-induced obesity in animal models: Points to consider and influence on metabolic markers. Diabetol. Metab. Syndr..

[6] Goodpaster B.H., Sparks L.M. (2017). Metabolic Flexibility in Health and Disease. Cell Metab..

[7] Smith R.L., Soeters M.R., Wust R.C.I., Houtkooper R.H. (2018). Metabolic Flexibility as an Adaptation to Energy Resources and Requirements in Health and Disease. Endocr. Rev..

[8] Fulghum K., Salathe S.F., Davis X., Thyfault J.P., Puchalska P., Crawford P.A. (2024). Ketone body metabolism and cardiometabolic implications for cognitive health. npj Metab. Health Dis..

[9] Sears B., Perry M. (2015). The role of fatty acids in insulin resistance. Lipids Health Dis..

[10] Hengist A., Davies R.G., Walhin J.P., Buniam J., Merrell L.H., Rogers L., Bradshaw L., Moreno-Cabanas A., Rogers P.J., Brunstrom J.M. (2024). Ketogenic diet but not free-sugar restriction alters glucose tolerance, lipid metabolism, peripheral tissue phenotype, and gut microbiome: RCT. Cell Rep. Med..

[11] Emanuele F., Biondo M., Tomasello L., Arnaldi G., Guarnotta V. (2025). Ketogenic Diet in Steatotic Liver Disease: A Metabolic Approach to Hepatic Health. Nutrients.

[12] Puchalska P., Crawford P.A. (2021). Metabolic and Signaling Roles of Ketone Bodies in Health and Disease. Annu. Rev. Nutr..

[13] Newman J.C., Verdin E. (2014). Ketone bodies as signaling metabolites. Trends Endocrinol. Metab..

[14] Garcia-Rodriguez D., Gimenez-Cassina A. (2021). Ketone Bodies in the Brain Beyond Fuel Metabolism: From Excitability to Gene Expression and Cell Signaling. Front. Mol. Neurosci..

[15] Cahill G.F. (2006). Fuel metabolism in starvation. Annu. Rev. Nutr..

[16] McGarry J., Wright P.H., Foster D.W. (1975). Hormonal control of ketogenesis. Rapid activation of hepatic ketogenic capacity in fed rats by anti-insulin serum and glucagon. J. Clin. Investig..

[17] Fukao T., Song X.Q., Mitchell G.A., Yamaguchi S., Sukegawa K., Orii T., Kondo N. (1997). Enzymes of ketone body utilization in human tissues: Protein and messenger RNA levels of succinyl-coenzyme A (CoA):3-ketoacid CoA transferase and mitochondrial and cytosolic acetoacetyl-CoA thiolases. Pediatr. Res..

[18] Cheng C.W., Biton M., Haber A.L., Gunduz N., Eng G., Gaynor L.T., Tripathi S., Calibasi-Kocal G., Rickelt S., Butty V.L. (2019). Ketone Body Signaling Mediates Intestinal Stem Cell Homeostasis and Adaptation to Diet. Cell.

[19] Balasse E.O., Fery F. (1989). Ketone body production and disposal: Effects of fasting, diabetes, and exercise. Diabetes Metab. Rev..

[20] Newman J.C., Verdin E. (2017). beta-Hydroxybutyrate: A Signaling Metabolite. Annu. Rev. Nutr..

[21] Hemingway C., Freeman J.M., Pillas D.J., Pyzik P.L. (2001). The ketogenic diet: A 3- to 6-year follow-up of 150 children enrolled prospectively. Pediatrics.

[22] Beckett T.L., Studzinski C.M., Keller J.N., Paul Murphy M., Niedowicz D.M. (2013). A ketogenic diet improves motor performance but does not affect beta-amyloid levels in a mouse model of Alzheimer’s disease. Brain Res..

[23] Prins M.L., Lee S.M., Fujima L.S., Hovda D.A. (2004). Increased cerebral uptake and oxidation of exogenous betaHB improves ATP following traumatic brain injury in adult rats. J. Neurochem..

[24] Yurista S.R., Nguyen C.T., Rosenzweig A., de Boer R.A., Westenbrink B.D. (2021). Ketone bodies for the failing heart: Fuels that can fix the engine?. Trends Endocrinol. Metab..

[25] Dmitrieva-Posocco O., Wong A.C., Lundgren P., Golos A.M., Descamps H.C., Dohnalova L., Cramer Z., Tian Y., Yueh B., Eskiocak O. (2022). beta-Hydroxybutyrate suppresses colorectal cancer. Nature.

[26] Ang Q.Y., Alexander M., Newman J.C., Tian Y., Cai J., Upadhyay V., Turnbaugh J.A., Verdin E., Hall K.D., Leibel R.L. (2020). Ketogenic Diets Alter the Gut Microbiome Resulting in Decreased Intestinal Th17 Cells. Cell.

[27] Li X., Yang J., Zhou X., Dai C., Kong M., Xie L., Liu C., Liu Y., Li D., Ma X. (2024). Ketogenic diet-induced bile acids protect against obesity through reduced calorie absorption. Nat. Metab..

[28] Zhu H., Bi D., Zhang Y., Kong C., Du J., Wu X., Wei Q., Qin H. (2022). Ketogenic diet for human diseases: The underlying mechanisms and potential for clinical implementations. Signal Transduct. Target. Ther..

[29] Rynders C.A., Thomas E.A., Zaman A., Pan Z., Catenacci V.A., Melanson E.L. (2019). Effectiveness of Intermittent Fasting and Time-Restricted Feeding Compared to Continuous Energy Restriction for Weight Loss. Nutrients.

[30] Vasim I., Majeed C.N., DeBoer M.D. (2022). Intermittent Fasting and Metabolic Health. Nutrients.

[31] Muscella A., Stefano E., Lunetti P., Capobianco L., Marsigliante S. (2020). The Regulation of Fat Metabolism During Aerobic Exercise. Biomolecules.

[32] Storoschuk K.L., Wood T.R., Stubbs B.J. (2023). A systematic review and meta-regression of exogenous ketone infusion rates and resulting ketosis-A tool for clinicians and researchers. Front. Physiol..

[33] Wang Q., Zhou Y., Rychahou P., Fan T.W., Lane A.N., Weiss H.L., Evers B.M. (2017). Ketogenesis contributes to intestinal cell differentiation. Cell Death Differ..

[34] Sun F., Wang K., Dong X., Secaira-Morocho H., Hui A., Cai C., Sze J.J., Low B., Udgata S., Pasch C.A. (2025). The Microbial Bile Acid Metabolite 3-oxo-LCA Inhibits Colorectal Cancer Progression. Cancer Res..

[35] Dong X., Sun F., Secaira-Morocho H., Hui A., Wang K., Cai C., Udgata S., Low B., Wei S., Chen X. (2024). The dichotomous roles of microbial-modified bile acids 7-oxo-DCA and isoDCA in intestinal tumorigenesis. Proc. Natl. Acad. Sci. USA.

[36] Moro C., Magnan C. (2025). Revisited guidelines for metabolic tolerance tests in mice. Lab Anim..

[37] Wu Y., Li B., Li L., Mitchell S.E., Green C.L., D’Agostino G., Wang G., Wang L., Li M., Li J. (2021). Very-low-protein diets lead to reduced food intake and weight loss, linked to inhibition of hypothalamic mTOR signaling, in mice. Cell Metab..

[38] Augustin K., Khabbush A., Williams S., Eaton S., Orford M., Cross J.H., Heales S.J.R., Walker M.C., Williams R.S.B. (2018). Mechanisms of action for the medium-chain triglyceride ketogenic diet in neurological and metabolic disorders. Lancet Neurol..

[39] Kosinski C., Jornayvaz F.R. (2017). Effects of Ketogenic Diets on Cardiovascular Risk Factors: Evidence from Animal and Human Studies. Nutrients.

[40] Kong Y.W., Morrison D., Lu J.C., Lee M.H., Jenkins A.J., O’Neal D.N. (2024). Continuous ketone monitoring: Exciting implications for clinical practice. Diabetes Obes. Metab..

[41] Crosier R., McPherson R. (2022). Profound Elevation in LDL Cholesterol Level Following a Ketogenic Diet: A Case Series. CJC Open.

[42] Bueno N.B., de Melo I.S., de Oliveira S.L., da Rocha Ataide T. (2013). Very-low-carbohydrate ketogenic diet v. low-fat diet for long-term weight loss: A meta-analysis of randomised controlled trials. Br. J. Nutr..

[43] Cuenoud B., Hartweg M., Godin J.P., Croteau E., Maltais M., Castellano C.A., Carpentier A.C., Cunnane S.C. (2020). Metabolism of Exogenous D-Beta-Hydroxybutyrate, an Energy Substrate Avidly Consumed by the Heart and Kidney. Front. Nutr..

