# Supplementary material for: Minimum Dietary Fat Threshold for Effective Ketogenesis and Obesity Control in Mice
Source: Nutrients. 2025 Oct 12;17(20):3203. doi: 10.3390/nu17203203 (PMC12567501; doi:10.3390/nu17203203)
Supplement: Supplementary file 1 [file nutrients-17-03203-s001.zip › Supplementary Table S2_workflow.pdf]

Scheme 1	
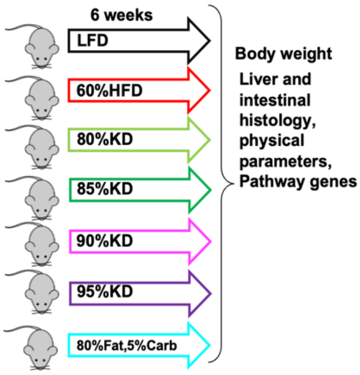	Figure 1<br>Figure 2<br>Figure 3<br>Figure S1<br>Figure S2

Scheme 2	
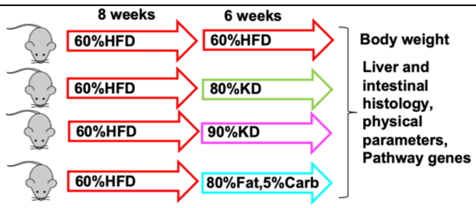	Figure 4<br>Figure 5<br>Figure 6<br>Figure S3<br>Figure S4
