# Supplementary material for: Minimum Dietary Fat Threshold for Effective Ketogenesis and Obesity Control in Mice
Source: Nutrients. 2025 Oct 12;17(20):3203. doi: 10.3390/nu17203203 (PMC12567501; doi:10.3390/nu17203203)
Supplement: Supplementary file 1 [file nutrients-17-03203-s001.zip › Supplementary Table S3.pdf]

Supplementary Table S3

| Primer | Sequence |
|---|---|
| 36B4-F | ACCTCCTTCTTCTTCCAGGCTTT |
| 36B4-R | CCCACCTTGTCTCCAGTCTTT |
| Cpt1a-F | CTCCGCCTGAGCCATGAAG |
| Cpt1a-R | CACCAGTGATGATGCCATTCT |
| Cd36-F | GCGACATGATTAATGGCACA |
| Cd36-R | CCTGCAAATGTCAGAGGAAA |
| Acat1-F | CAGGAAGTAAGATGCCTGGAAC |
| Acat1-R | TTCACCCCCTTGGATGACATT |
| Hmgcs2-F | ATACCACCAACGCCTGTTATGG |
| Hmgcs2-R | CAATGTCACCACAGACCACCAG |
| Hmgcl-F | CAGGTGAAGATCGTGGAAGTC |
| Hmgcl-R | GGAGCCCTGCTTCGGAAAG |
| Bdh1-F | TTCCTCTGTCATCAACGCTGTCAC |
| Bdh1-R | ATCCGAAGCCACCAGTAGTAGTCC |
| Mct1-F | TCAGTGCAACGACCAGTGAAGTATC |
| Mct1-R | AAGCCGCAACCAGACAGACAAC |
| Oxct1-F | GGACGGCATGTACGCTAACTTGG |
| Oxct1-R | GCTTCGTCTTTCAGTGGGTATGGG |
| Cyp7a1-F | CATCTCAAGCAAACACCATTCC |
| Cyp7a1-R | TCACTTCTTCAGAGGCTGGTTTC |
| Cyp8b1-F | GAACTCAACCAGGCCATGCT |
| Cyp8b1-R | AGGAGCTGGCACCTAGACT |
| Cyp27a1-F | GAGAGTGAATCAGGGGACCA |
| Cyp27a1-R | TCAGGAATGGAGGGTTTCAG |
| Cyp7b1-F | CCTTGTGAACCACCCTTGAT |
| Cyp7b1-R | GTGTCACCATGTTGCCTTTG |
| Fxr-F | CAGACAGCTAATGAGGACGACA |
| Fxr-R | CCGTGAGTTCCGTTTTCTCC |
| Shp-F | CTACCCTCAAGAACATTCCAGG |
| Shp-R | CACCAGACTCCATTCCACG |
| Ibabp-F | CACCATTGGCAAAGAATGTG |
| Ibabp-R | AACTTGTCACCCACGACCTC |
| Ost$\alpha$-F | TACAAGAACACCCTTTGCCC |
| Ost$\alpha$-R | AGGAATCCAGAGACCAAAGC |
| Ost$\beta$-F | GTATTTTCGTGCAGAAGATGC |
| Ost$\beta$-R | ATTTCTGTTTGCCAGGATGCT |
| Asbt-F | GTCTGTCCCCCAAATGCAACT |
| Asbt-R | CACCCCATAGAAAACATCACC |
| Fasn-F | AAGTTGCCCGAGTCAGAGAACC |
| Fasn-R | ATCCATAGAGCCCAGCCTTCCATC |
