# Supplementary figures and images for: Minimum Dietary Fat Threshold for Effective Ketogenesis and Obesity Control in Mice
Source: Nutrients. 2025 Oct 12;17(20):3203. doi: 10.3390/nu17203203 (PMC12567501; doi:10.3390/nu17203203)

**Sup Figure S1, the rest data of Figure 1 n 2 n 3**

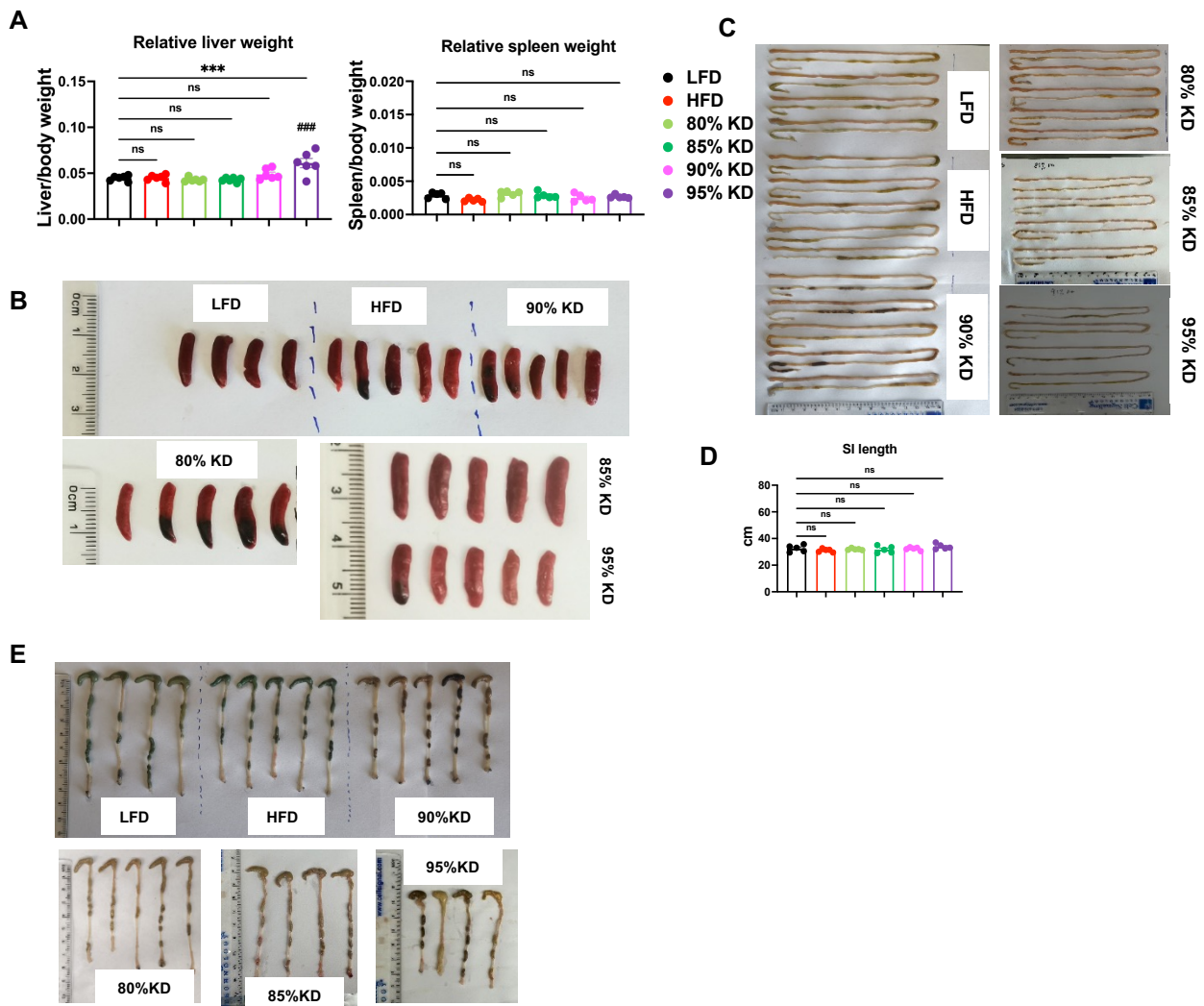

Supplement: Supplementary file 1 [file nutrients-17-03203-s001.zip › Supplementary Figure S1.pdf]

Sup Figure S2 (80%+5%)

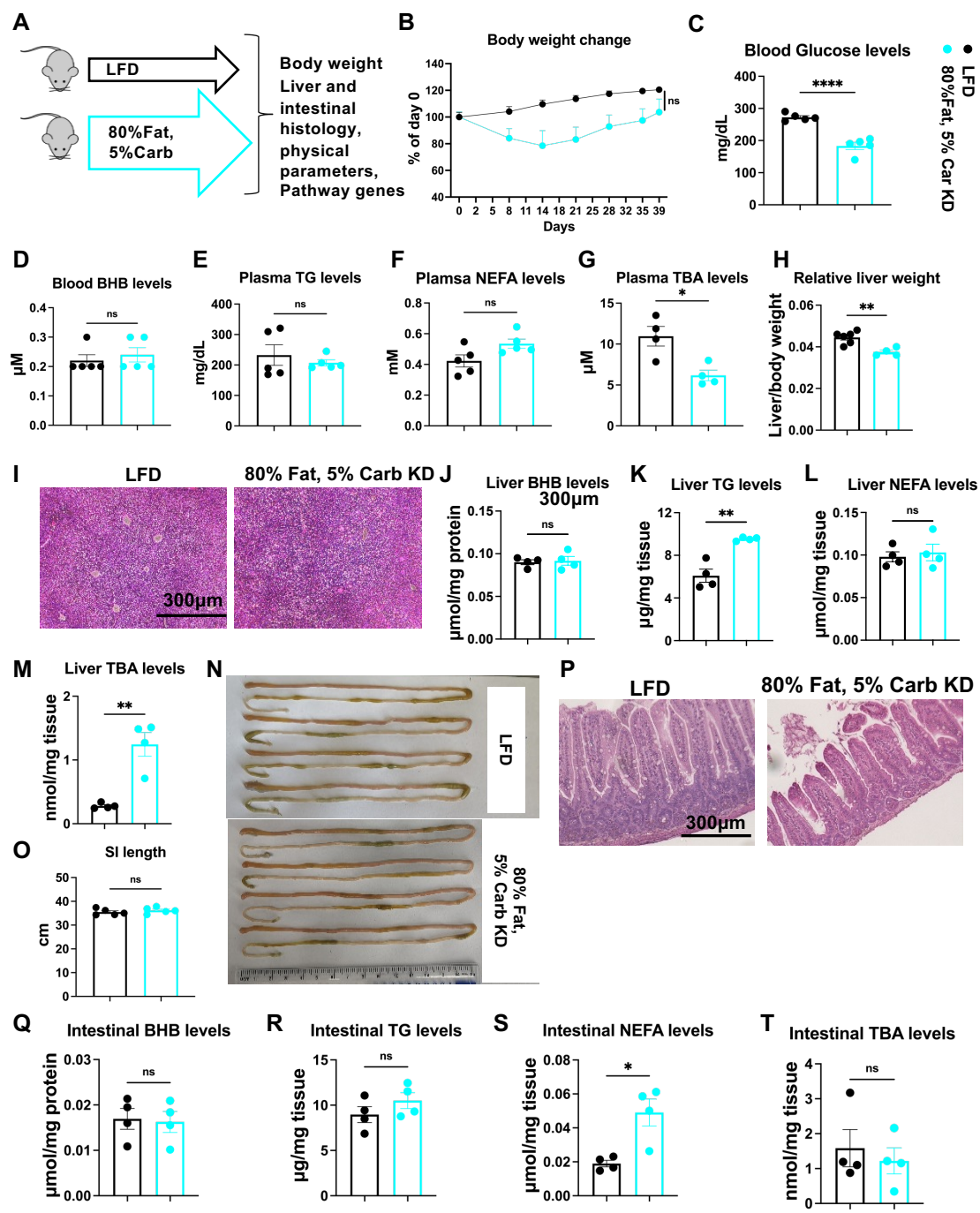

Supplement: Supplementary file 1 [file nutrients-17-03203-s001.zip › Supplementary Figure S2.pdf]

Sup Figure S3, the rest data of Figure 4 n 5

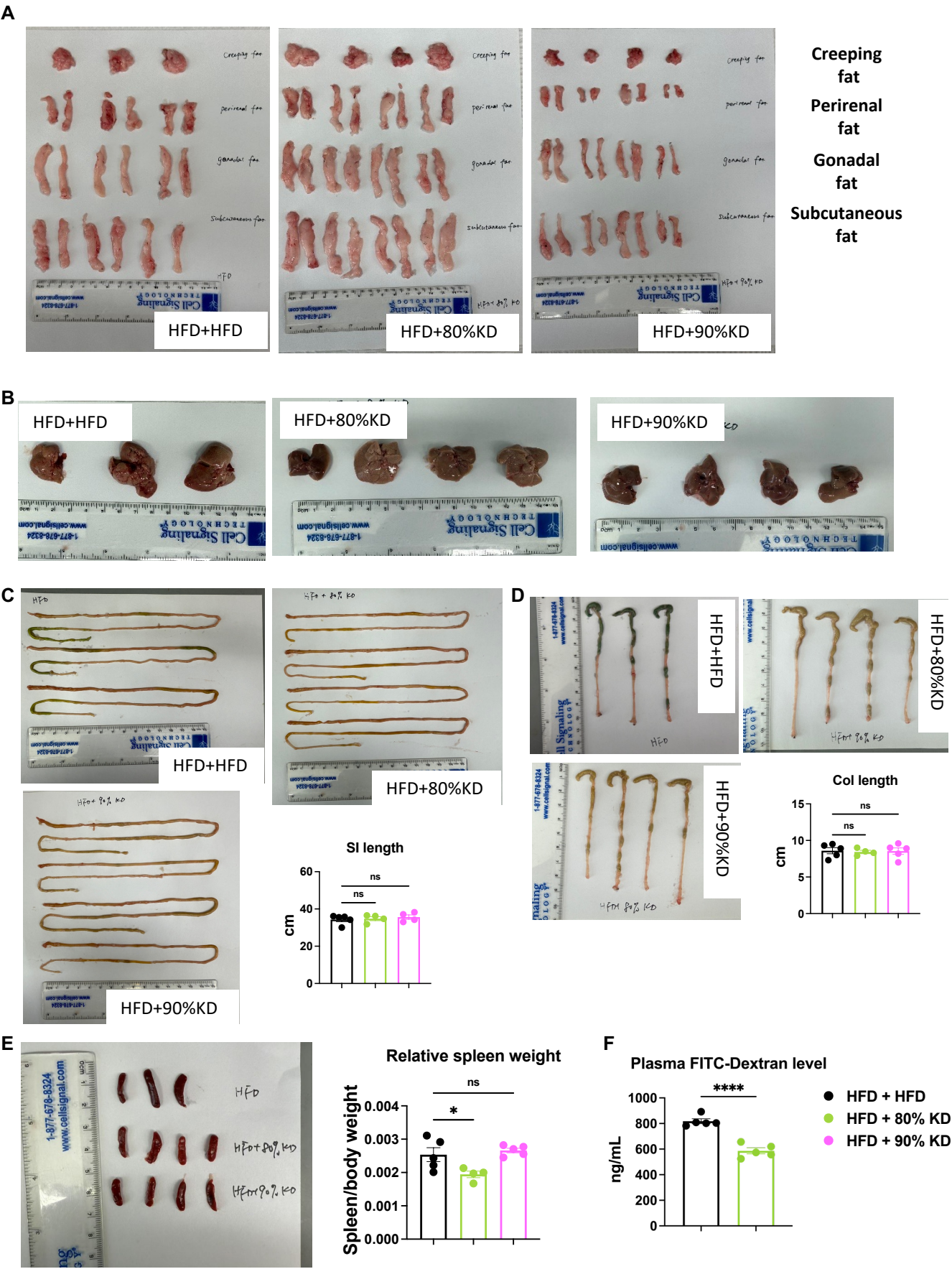

Supplement: Supplementary file 1 [file nutrients-17-03203-s001.zip › Supplementary Figure S3.pdf]

Sup Figure S4, HFD+80% Fat, 5% Carb KD

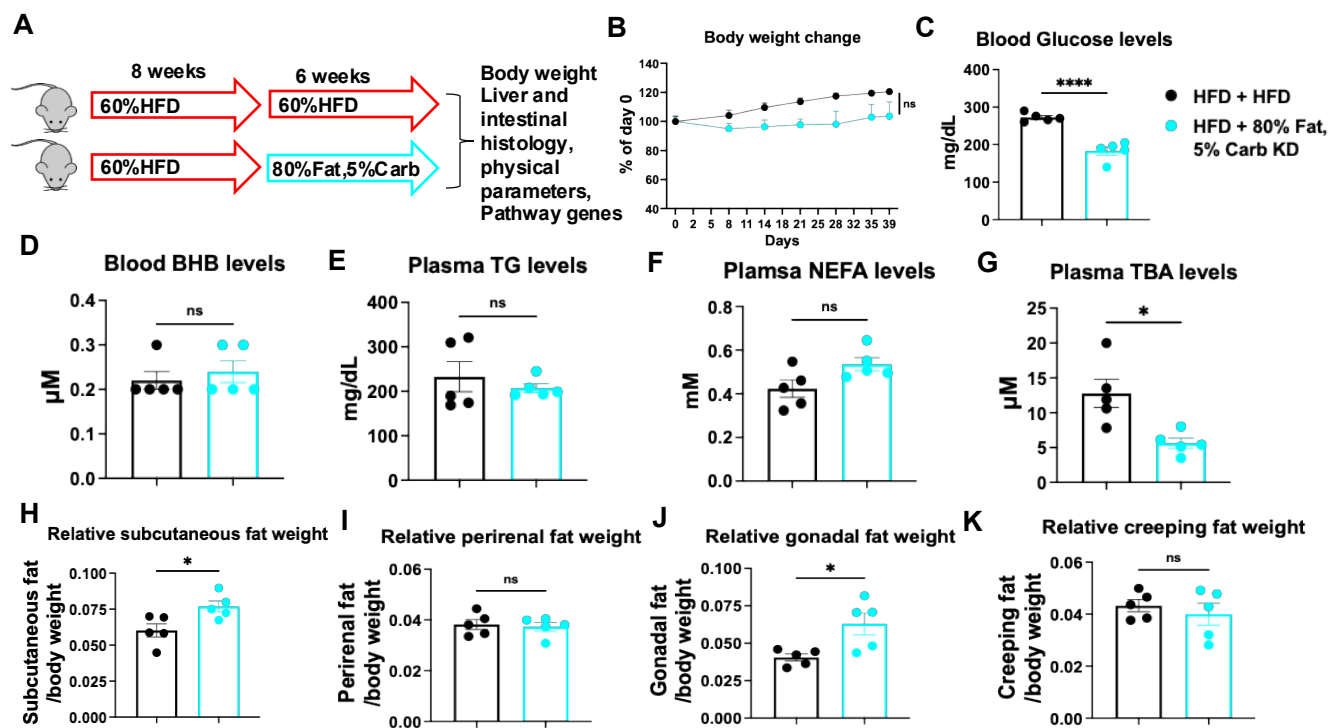

Supplement: Supplementary file 1 [file nutrients-17-03203-s001.zip › Supplementary Figure S4.pdf]
